# Expression of turtle riboflavin-binding protein represses mitochondrial electron transport gene expression and promotes flowering in Arabidopsis

**DOI:** 10.1186/s12870-014-0381-5

**Published:** 2014-12-30

**Authors:** Liang Li, Li Hu, Li-Ping Han, Hongtao Ji, Yueyue Zhu, Xiaobing Wang, Jun Ge, Manyu Xu, Dan Shen, Hansong Dong

**Affiliations:** Department of Plant Pathology, Nanjing Agricultural University and State Ministry of Education Key Laboratory of Integrated Management of Crop Pathogens and Insect Pests, Nanjing, 210095 China

## Abstract

**Background:**

Recently we showed that *de novo* expression of a turtle riboflavin-binding protein (RfBP) in transgenic Arabidopsis increased H_2_O_2_ concentrations inside leaf cells, enhanced the expression of floral regulatory gene *FD* and floral meristem identity gene *AP1* at the shoot apex, and induced early flowering. Here we report that RfBP-induced H_2_O_2_ presumably results from electron leakage at the mitochondrial electron transport chain (METC) and this source of H_2_O_2_ contributes to the early flowering phenotype.

**Results:**

While enhanced expression of *FD* and *AP1* at the shoot apex was correlated with early flowering, the foliar expression of 13 of 19 METC genes was repressed in RfBP-expressing (RfBP^+^) plants. Inside RfBP^+^ leaf cells, cytosolic H_2_O_2_ concentrations were increased possibly through electron leakage because similar responses were also induced by a known inducer of electron leakage from METC. Early flowering no longer occurred when the repression on METC genes was eliminated by *RfBP* gene silencing, which restored RfBP^+^ to wild type in levels of *FD* and *AP1* expression, H_2_O_2_, and flavins. Flowering was delayed by the external riboflavin application, which brought gene expression and flavins back to the steady-state levels but only caused 55% reduction of H_2_O_2_ concentrations in RfBP^+^ plants. RfBP-repressed METC gene expression remedied the cytosolic H_2_O_2_ diminution by genetic disruption of transcription factor NFXLl and compensated for compromises in *FD* and *AP1* expression and flowering time. By contrast, RfBP resembled a peroxisomal catalase mutation, which augments the cytosolic H_2_O_2_, to enhance *FD* and *AP1* expression and induce early flowering.

**Conclusions:**

RfBP-repressed METC gene expression potentially causes electron leakage as one of cellular sources for the generation of H_2_O_2_ with the promoting effect on flowering. The repressive effect on METC gene expression is not the only way by which RfBP induces H_2_O_2_ and currently unappreciated factors may also function under RfBP^+^ background.

**Electronic supplementary material:**

The online version of this article (doi:10.1186/s12870-014-0381-5) contains supplementary material, which is available to authorized users.

## Background

Riboflavin (vitamin B_2_) is the precursor of flavin mononucleotide (FMN) and flavin adenine dinucleotide (FAD), essential cofactors for many metabolic enzymes involved in multiple cellular processes, such as mitochondrial electron transport chain (METC) and cellular redox regulation in other cellular compartments [1-3]. Flavin-mediated redox is critical for the generation of reactive oxygen species (ROS) of different types [4-6], such as superoxide radical O_2_^•–^ [7,8] and hydrogen peroxide H_2_O_2_ [4,9]. H_2_O_2_ is a more stable ROS form, than O_2_^•–^ for example, and thus frequently functions as a cellular signal to regulate multiple aspects of plant development [10,11].

ROS can be generated by a number of redox processes outside and inside plant cells [9,11-13]. An intracellular source of ROS is redox-associated electron-carrier protein complexes I to IV in METC [14]. If METC functions normally, an electron tetrad (four electrons as a group) in each transport round is transferred through the carrier-protein complexes to a single O_2_ accepter, which reduces O_2_ to form H_2_O with protons from coenzymes NADH_2_ (nicotinamide adenine dinucleotide carrying two protons) and FADH_2_ [15-17]. Under METC dysfunction, single electrons are transferred to O_2_ to generate O_2_^•–^, which is further converted to H_2_O_2_ [18-21]. This process is known as electron leakage and increases cytosolic concentrations of H_2_O_2_ through subcellular trafficking [11,13]. Electron leakage and H_2_O_2_ generation may take place in protein complexes I, II, and III in living organisms including plants [22-25]. Electron leakage and H_2_O_2_ generation subsequent to complex I inhibition by rotenone, a ketonic chemical compound that interferes with METC, have been well demonstrated in animals [20,21]. Because FMN/FMNH_2_ and FAD/FADH_2_ serve as redox centers in complexes I and II, respectively, flavins are likely to play a pivotal role in electron leakage and H_2_O_2_ generation from METC [13,21,26].

In agreement with this notion, recently we demonstrated that cell cytosolic H_2_O_2_ concentrations could be altered by modulating concentrations of free flavins (riboflavin, FMN, and FAD) in leaves of *Arabidopsis thaliana* [13]. Flavin concentrations were modulated by *de novo* expression of the turtle (*Trionyx sinensis japonicus*) gene encoding riboflavin-binding protein (RfBP). This protein contains a nitroxyl-terminal ligand-binding domain, which is implicated in molecular interactions, and a carboxyl-terminal phosphorylation domain, which accommodates the riboflavin molecule [27-30]. In the *RfBP*-expressing (RfBP^+^) Arabidopsis plants, RfBP localizes to chloroplasts and binds with riboflavin, resulting in significant decreases of free flavin concentrations. This change accompanies an elevation in the cytosolic level of H_2_O_2_. All these RfBP-conferred responses can be eliminated by nullifying RfBP production under RfBP^+^ background, and the *RfBP* gene silencing (RfBP^−^) Arabidopsis lines resemble the wild-type (WT) plant in flavin and H_2_O_2_ concentrations [13]. Thus, the alteration of flavin content is an initial force for H_2_O_2_ generation in the plant cytosol. Nevertheless, how altered flavin content induces H_2_O_2_ generation was unclear.

H_2_O_2_ has been implicated in flowering time control [31-35] by the photoperiod pathway, which comprises a number of regulators [36,37]. An essential regulator, the bZIP transcription factor FLOWERING LOCUS D (FD), functions to activate the floral meristem identity (FMI) gene *APETALA1* (*AP1*), which marks the beginning of floral organ formation at the shoot apex [38,39]. At the shoot apex, *FD* and *AP1* are coordinately expressed to promote the growth of floral organ primordia [38,39]. The circadian clock is a central player of the photoperiod pathway [36], and H_2_O_2_ serves as an input signal that affects the transcriptional output of the clock and flowering time [35]. Flowering is promoted when the cytosolic H_2_O_2_ level is increased, for example, by enhanced activities of chloroplastic lipoxygenase and ascorbate peroxidase in Arabidopsis [31,32].

In addition to increasing H_2_O_2_, downregulation of leaf flavin content by RfBP also induces early flowering in relation to enhanced expression of floral promoting genes [13,40]. Early flowering was a serendipitous phenomenon [13] and was prudently characterized as a constant phenotype of RfBP^+^ plants [40]. This phenotype was eliminated when leaf flavins were brought back by RfBP^**−**^ to the steady-state levels. RfBP-induced early flowering was correlated with enhanced foliar expression of floral promoting photoperiod genes, but not related to genes in vernalization, autonomous, and gibberellin pathways [40], which provide flowering regulation mechanisms alternative to the photoperiod [41-43]. RfBP-upregulated photoperiod genes encode red/far red light receptor phytochrome PHYA, blue light receptor cryptochromes CRY1 and CRY2, circadian clock oscillator TIMING OF CAB EXPRESSION1 (TOC1), and putative zinc finger transcription factor CONSTANS (CO) proteins [40]. PHYA, CRY1, and CRY2 serve as the entry of the clock and transmit the light signal to the central oscillator, which deploys a *TOC1*-partnering transcriptional feedback loop to control day-night rhythm of photoperiod gene expression [44-46] and the production of CO as an output of the clock and an activator of the florigen gene *FT* in leaves [45,47]. Thus, RfBP-induced early flowering is attributable to the photoperiod pathway. RfBP-induced early flowering also correlates with increased expression of *FD* and *AP1* at the shoot apex [40], suggesting the role of RfBP in concurrently enhancing the expression of flowering-related genes assigned to photoperiod, floral regulation, and FMI categories. By contrast, the expression of *FT* and photoperiod genes in leaves and the expression of *FD* and *AP1* in the shoot apex were no longer enhanced when the *RfBP* gene was silenced, RfBP protein production canceled, and flavin concentrations were brought back to the steady-state levels [40], confirming the initial effects of RfBP modulation on the sequential responses. These findings indicate that leaf flavin content downregulation by RfBP induces early flowering coincidently with increased content of cytosolic H_2_O_2_ and enhanced expression of genes that promote flowering through the photoperiod pathway. However, causal relationships of these responses were unknown. Here, we focus on a particular question: how is H_2_O_2_ induced to affect flowering time under RfBP^+^ background?

In the plant cell, H_2_O_2_ can be generated by multiple sources, such as peroxisomal redox [48,49], chloroplastic metabolisms [31,32], transcriptional regulation related to growth and development [50], and METC as well [11,13]. However, which of these sources is related to flowering time control was unknown. In this study, we elucidate that leaf flavin content downregulation by RfBP [13,40] induces H_2_O_2_ generation presumably through electron leakage from METC and this source of H_2_O_2_ causes a promoting effect on flowering in Arabidopsis.

## Results

### RfBP induces early flowering and expression of *FD* and *AP1* genes

**Figure 1:**
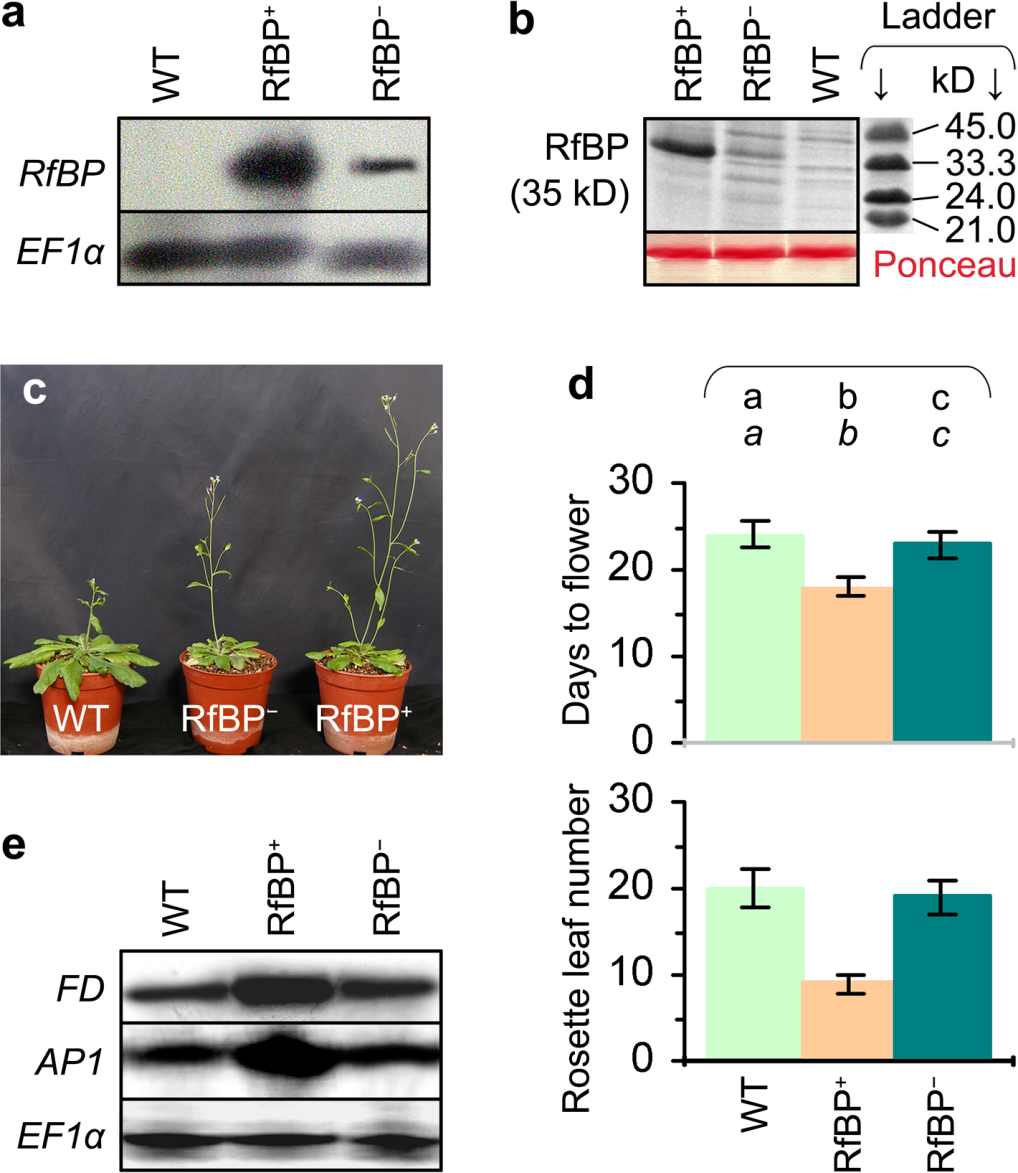
***De novo*** **expression of the turtle** ***RfBP*** **gene and its effects on flowering and expression of** ***FD*** **and** ***AP1*** **genes in Arabidopsis.** WT, RfBP^+^, and RfBP^−^ plants were grown in long days. Northern blotting **(a)** and electrophoresis **(b)** analyses were performed with RNAs and proteins, respectively, isolated from the two youngest expanded leaves of 12-day-old plants. Gel staining with in **(b)** verified consistent loading of proteins. Three-week-old plants were photographed **(c)**. Days to flower and rosette leaf number were scored as mean values ± standard deviations from seven experimental repeats each containing 50 plants **(d)**. On bar graphs, different letters shown in regular and *italic* fonts indicate significant differences by analysis of variance using Fisher’s least significant difference test and Tukey-Kramer’s test, respectively (n = 7; *P* < 0.01). *FD* and *AP1* were analyzed by Northern blotting with RNAs from shoot apices of 12-day-old plants **(e)**. In **(a)** and **(e)**, the constitutively expressed *EF1α* gene was used as a reference.

Previously we tested WT, RfBP^+^, and RfBP^−^ plants under typical short days (8-hour light), atypical short days (12 hours), typical long days (16 hours), or inductive photoperiod (plant shift from short days to long days ) [13,40]. To simplify experimental conditions in this study, we investigated those plants grown in typical long days and under this condition we confirmed *de novo* expression of the *RfBP* gene in RfBP^+^ and gene silencing in RfBP^−^. The gene was highly expressed (Figure 1a) and a substantial quantity of the RfBP protein was produced (Figure 1b) in leaves of RfBP^+^ in contrast to the absence of gene expression and protein production in the WT plant. The gene expression and protein production were markedly reduced in the RfBP^−^ plant (Figure 1a,b). Flowering was promoted in RfBP^+^ compared to WT or RfBP^−^ plants (Figure 1c). WT plants needed 24 days to flower with 20 rosette leaves (Figure 1d). RfBP^−^ resembled WT in flowering time and rosette leaf number while RFBP^+^ flowered 6 days earlier with a reduction of 11 rosette leaves than WT (Figure 1d). Then, we studied the floral initiation marker gene *AP1* and its regulator gene *FD* because enhanced expression of both genes well reflects the molecular basis of RfBP-induced early flowering [40]. We found that *FD* and *AP1* displayed higher expression levels in RfBP^+^ than in WT and RfBP^−^ plants on 12 days after stratification, 6 days before RfBP^+^ flowering in typical long days (Figure 1e). Therefore, it is pertinent that we further explore the molecular mechanism that underpins RfBP-induced early flowering under typical long day condition.

### Flavin downregulation by RfBP represses expression of METC genes

**Figure 2:**
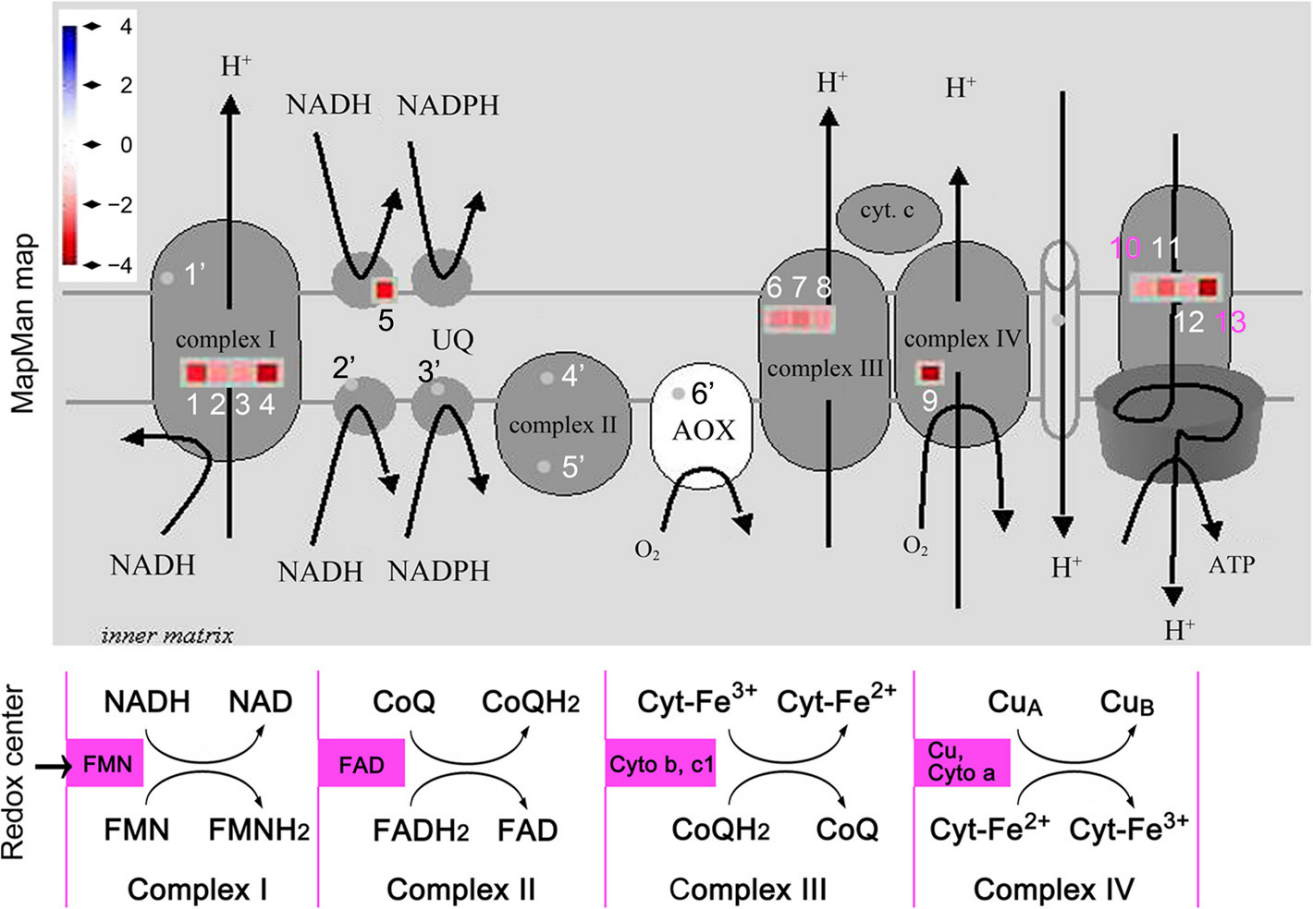
**The effect of RfBP on METC gene expression.** The MapMan program [85] was employed to analyze previously obtained data (http://www.ncbi.nlm.nih.gov/geo/query/acc.cgi?acc=GSE18417), show scaled reciprocal values of ratios of gene expression levels between RfBP^+^ and WT plants, and locate RfBP-affected genes with colored square patterns and other genes with grey dots in METC. Electron-carrier protein complexes and redox centers are indicated. In the MapMan map, RfBP-repressed genes are digitally coded (1–13) and the other genes are numbered with superscript commas. RfBP-repressed METC gene numbers 1–13 were used constantly in this figure and Figures 4, 5, and 10. See text for products encoded by METC genes.

Based on the RfBP-regulated transcriptome profiling by the Affymetrix Arabidopsis genome ATH1 array (http://www.ncbi.nlm.nih.gov/geo/query/acc.cgi?acc=GSE18417), expression levels of 13 of 19 METC genes were reduced 2 to 4 times in RfBP^+^ compared to the WT plant (Figure 2). The rest six genes encode: (1) NADH dehydrogenase (ubiquinone, CoQ) Fe-S protein; (2) iron-sulfur protein A; (3) iron-sulfur protein B; (4) iron-sulfur protein C; (5) flavoprotein and (6) alternative oxidase. Proteins encoded by RfBP-repressed METC genes in order are: (1) NADH-ubiquinone (NADHU) oxidoreductase-related,; (2) NADHU oxidoreductase-related; (3) NADHU oxidoreductase B18 subunit; (4) NADHU oxidoreductase 19-kD subunit (NDUFA8) family protein; (5) pridine nucleotide-disulphide oxidoreductase family protein; (6) ubiquinol-cytochrome (Cyt) c reductase (UCCR) complex 7.8-kD protein, putative; (7) putative UCCR complex CoQ-biding protein; (8) putative UCCR complex CoQ-biding protein; (9) Cyt c oxidase (UCCO) copper chaperone family protein; (10) UCCO subunit 6b, putative; (11) mitochondrial ATP synthase g subunit family protein; (12) mitochondrial ATP synthase g subunit family protein; and (13) mitochondrial ATP synthase episilon chain. In this list, the last three proteins function in the production of energy and the first 10 ones are all required for electron transport, initiated by NADH in complex I and finished by Cty in complex IV [16] (Figure 2).

**Figure 3:**
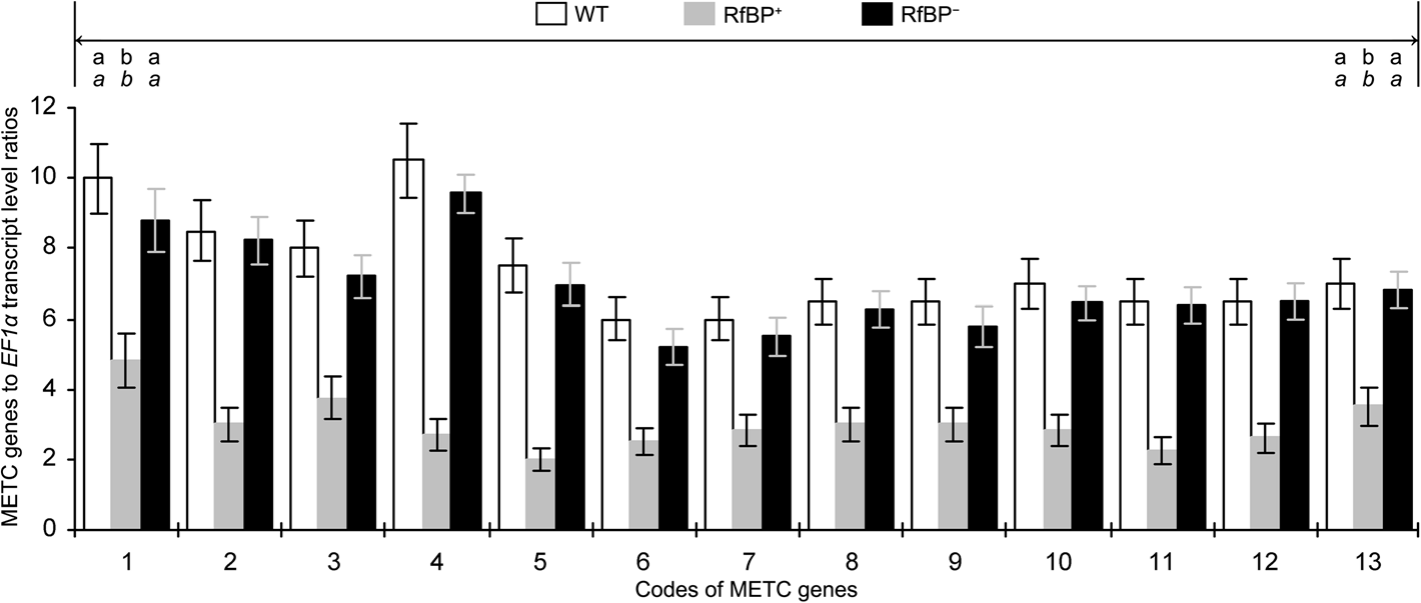
**Relative levels of METC gene expression in WT, RfBP** ^**+**^ **, and RfBP** ^**−**^ **plants.** Water and aqueous solutions of riboflavin and rotenone were used separately to immerse seeds and treat 10-day-old plants by spraying over plant tops. Gene expression in the two youngest expanded leaves of 12-day-old plants was analyzed by real-time RT-PCR using *EF1α* as a reference gene. Data shown are average values ± standard deviations of results from six experimental repeats each containing 15 individuals of 12-day-old plants. Different letters in regular and *italic* fonts indicate significant differences by analysis of variance using Fisher’s least significant difference test and Tukey-Kramer’s test, respectively (n = 6; *P* < 0.01), for every of 13 data pairs shown within the range of bidirectional arrowhead line.

The array result was confirmed by quantitative real-time RT-PCR analyses of gene expression in leaves. Based on ratios of transcript quantities to the constitutively expressed *EF1α* gene used as a reference, expression levels of the 13 METC genes were significantly (*P* < 0.01) lower in RfBP^+^ than in WT plants (Figure 3). The difference was more explicitly recognized by presentation of RfBP^+^ to WT ratios of gene transcript amounts (Additional file 1: Figure S1). Quantitative analyses did not detect evident repression of METC gene expression in RfBP^−^ plants. Instead, the 13 METC genes were expressed similarly in RfBP^−^ and WT leaves (Figure 3). This, repression of METC gene expression was caused by *de novo* expression of *RfBP*.

**Figure 4:**
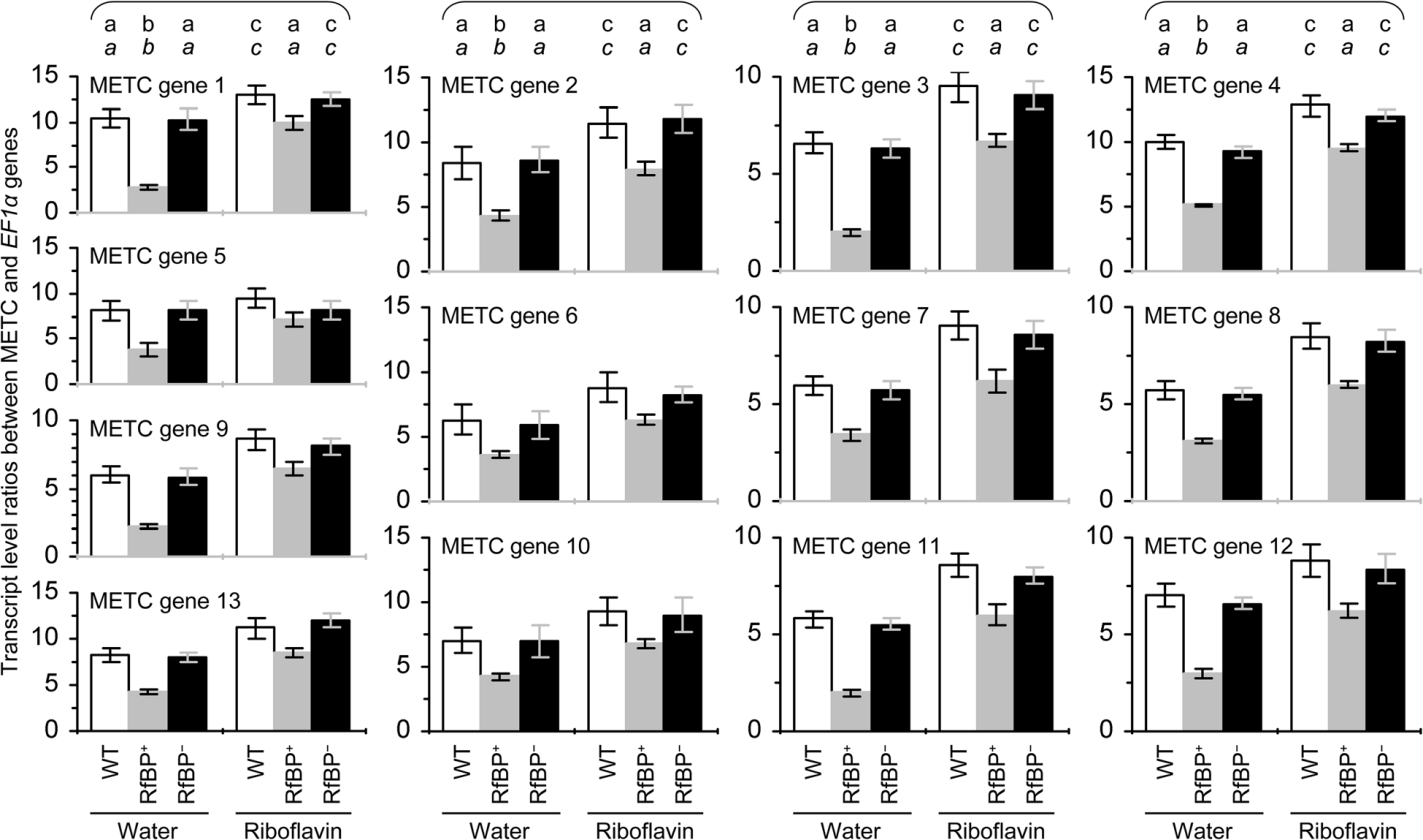
**Expression levels of METC genes in riboflavin-fed and water-treated WT, RfBP** ^**+**^ **, and RfBP** ^**−**^ **plants.** Water or an aqueous riboflavin solution was used to immerse seeds and treat 10-day-old plants by spraying over plant tops. Gene expression in the two youngest expanded leaves of 12-day-old plants was analyzed by real-time RT-PCR using *EF1α* as a reference gene. Ratios of transcript quantities between the tested METC genes and *EF1α* were quantified as mean values ± standard deviations from six experimental repeats each containing 15 plants. On bar graphs, different letters in regular and *italic* fonts indicate significant differences by analysis of variance using Fisher’s least significant difference test and Tukey-Kramer’s test, respectively (n = 6; *P* < 0.01).

**Figure 5:**
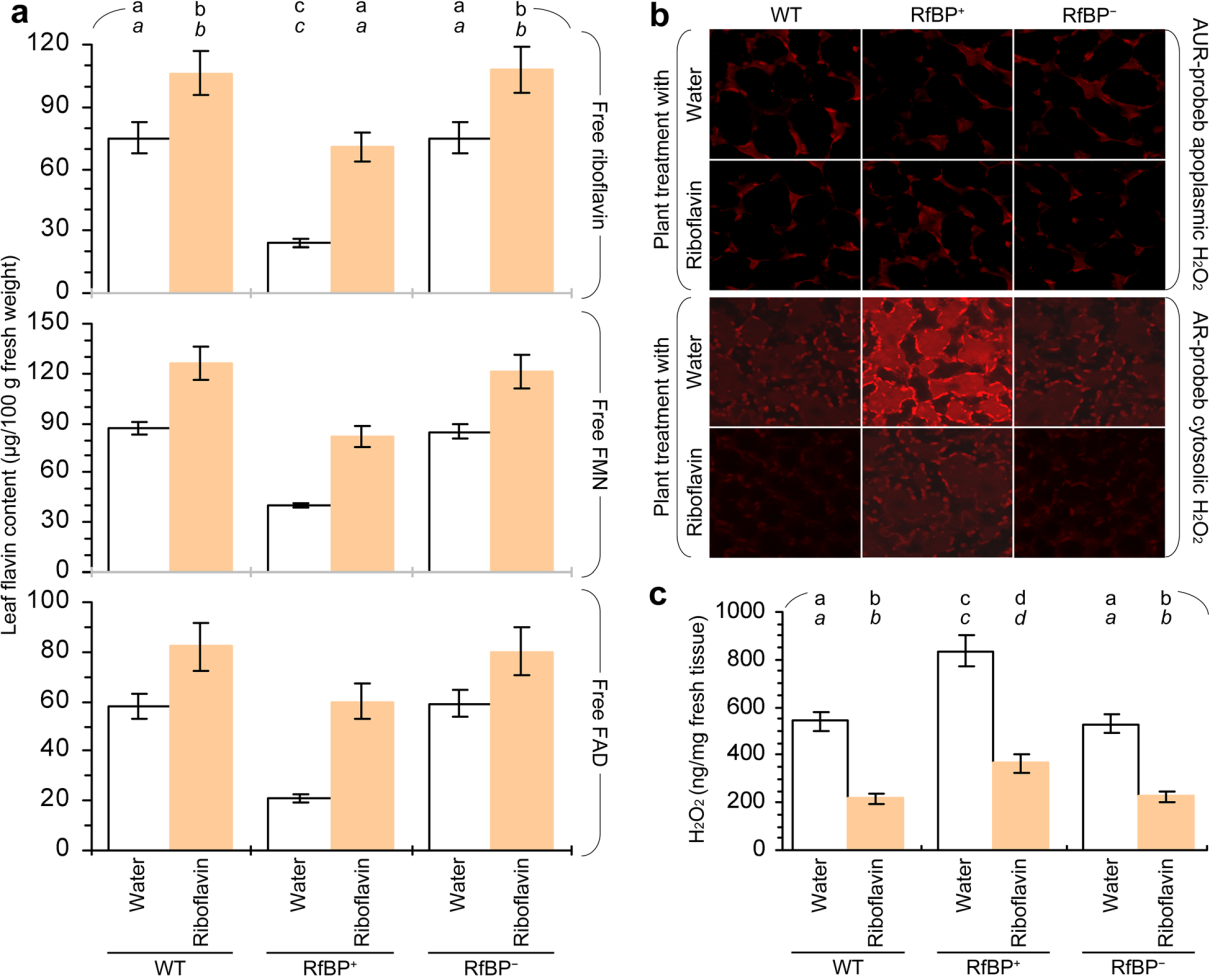
**Intrinsic flavins and H**_**2**_ **O**_**2**_ **in riboflavin-fed and water-treated plants.** Water or an aqueous solution of riboflavin was used to immerse seeds and treat 10-day-old plants by spraying over plant tops. Analyses for flavin concentrations **(a)**, subcellular H_2_O_2_ distribution **(b)**, and H_2_O_2_ concentrations **(c)** were performed on the two youngest leaves of 12-day-old plants. Quantitative data shown are average values ± standard deviations based on three experimental repeats each containing 15 plants. On bar graphs, different letters in regular and *italic* fonts indicate significant differences by analysis of variance using Fisher’s least significant difference test and Tukey-Kramer’s test, respectively (n = 3; *P* < 0.01).

We analyzed the relationship between the dual roles of RfBP in reducing METC gene expression and flavin concentrations. The 13 RfBP-repressed genes function in electron-carrier protein complexes I to IV while I and II employ FMN/FMNH_2_ and FAD/FADH_2_ as redox centers, respectively [14]. Thus, the suppression of METC gene expression might be attributed to flavin content reduction by RfBP. This hypothesis was validated by the pharmacological study in which plants were fed with an aqueous riboflavin solution or treated with water in the experimental control group. The 13 METC genes were expressed to greater extents in all plants following riboflavin feeding treatment compared to control, and in riboflavin-fed RfBP^+^ plants all of gene transcripts were retrieved approximately to the levels in water-treated WT plants (Figure 4). Meanwhile, the intrinsic flavin concentrations were increased in all plants following riboflavin feeding treatment, and flavin levels in riboflavin-fed RfBP^+^ plants were retrieved approximately to the steady-state level in water-treated WT plants (Figure 5a). RfBP^−^ performed similarly to WT in the riboflavin-feeding effect on flavin concentrations (Figure 5a). Based on statistical analyses, differences between RfBP^+^ and WT or RfBP^−^ plants in METC gene expression levels and the effects of riboflavin feeding treatment were constant and significant (*P* <0.01) for every gene (Figures 4 and 5a). Therefore, the suppression of METC gene expression is attributable to flavin content downregulation by RfBP.

### Repressed METC gene expression accompanies H_2_O_2_ generation presumably through electron leakage

As stated above, the repression of METC gene expression might impair METC functions and cause H_2_O_2_ generation through electron leakage. Electrons leak mainly from electron-carrier protein complex I or III and occasionally from complex II [24,25,51]. Because the redox center is FMN/FMNH_2_ in complex I and FAD/FADH_2_ in complex II ([14]; Figure 2), flavin content reduction by RfBP is likely to impair functions of both complexes and induce electron leakage. To verify this hypothesis, we tested H_2_O_2_ in leaves of WT, RfBP^+^, and RfBP^−^ plants following riboflavin feeding treatment since the treatment eliminated the inhibitive effect of RfBP on METC genes (Figure 4) and restores RfBP^+^ to WT in flowering time [40].

Fluorescent H_2_O_2_ probes Amplex red (AR) and Amplex ultra red (AUR) were employed to visualize H_2_O_2_ in Arabidopsis cells. In reaction with H_2_O_2_, AR and AUR are converted into resorufin and a resorufin analog, respectively, which emit strong crimson fluorescence [9]. AR can penetrate the plasma membrane and thus probes H_2_O_2_ in the cytosol, whereas, AUR can not penetrate the plasma membrane and thus probes H_2_O_2_ present in the apoplastic space [9]. Apoplastic and cytosolic H_2_O_2_ signals reported by AUR and AR, respectively, are shown in Figure 5b. AUR staining signals were weak and similar in all plants irrespectively of treatment with riboflavin or with water as a control, suggesting low steady-state levels of the apoplastic H_2_O_2_ that was unaffected by RfBP or riboflavin. By contrast, AR staining signals were stronger in all plants treated with water compared to riboflavin, suggesting that riboflavin feeding treatment decreased the quantity of cytosolic H_2_O_2_. Especially, RfBP^+^ plants displayed the strongest signal with water treatment but the signal was highly reduced by riboflavin feeding treatment. Thus, RfBP-induced H_2_O_2_ mainly accumulates in the cytosol and can be decreased by feeding plants with riboflavin.

Leaf H_2_O_2_ concentrations were measured. With water treatment, H_2_O_2_ levels were approximately 1.6-fold higher in RfBP^+^ than in WT or RfBP^−^ (Figure 5c). Riboflavin feeding treatment significantly (*P* < 0.01) decreased H_2_O_2_ concentrations in all plants. Unexpectedly, H_2_O_2_ concentrations in riboflavin-fed RfBP^+^ plants were reduced only by 55%, from 831 to 372 ng/mg fresh leaf weight, still significantly (*P* < 0.01) lower than in water-treated WT (573 ng/mg) or RfBP^−^ (530 ng/mg) plants (Figure 5c). In all cases, however, H_2_O_2_ and flavin levels (Figure 5a,b) were correlated with expression extents of METC genes (Figure 4). These analyses are in agreement with H_2_O_2_ imaging assays and both lines of evidence suggest the possibility that increased cytosolic H_2_O_2_ results from electron leakage in flavin-dependent METC.

**Figure 6:**
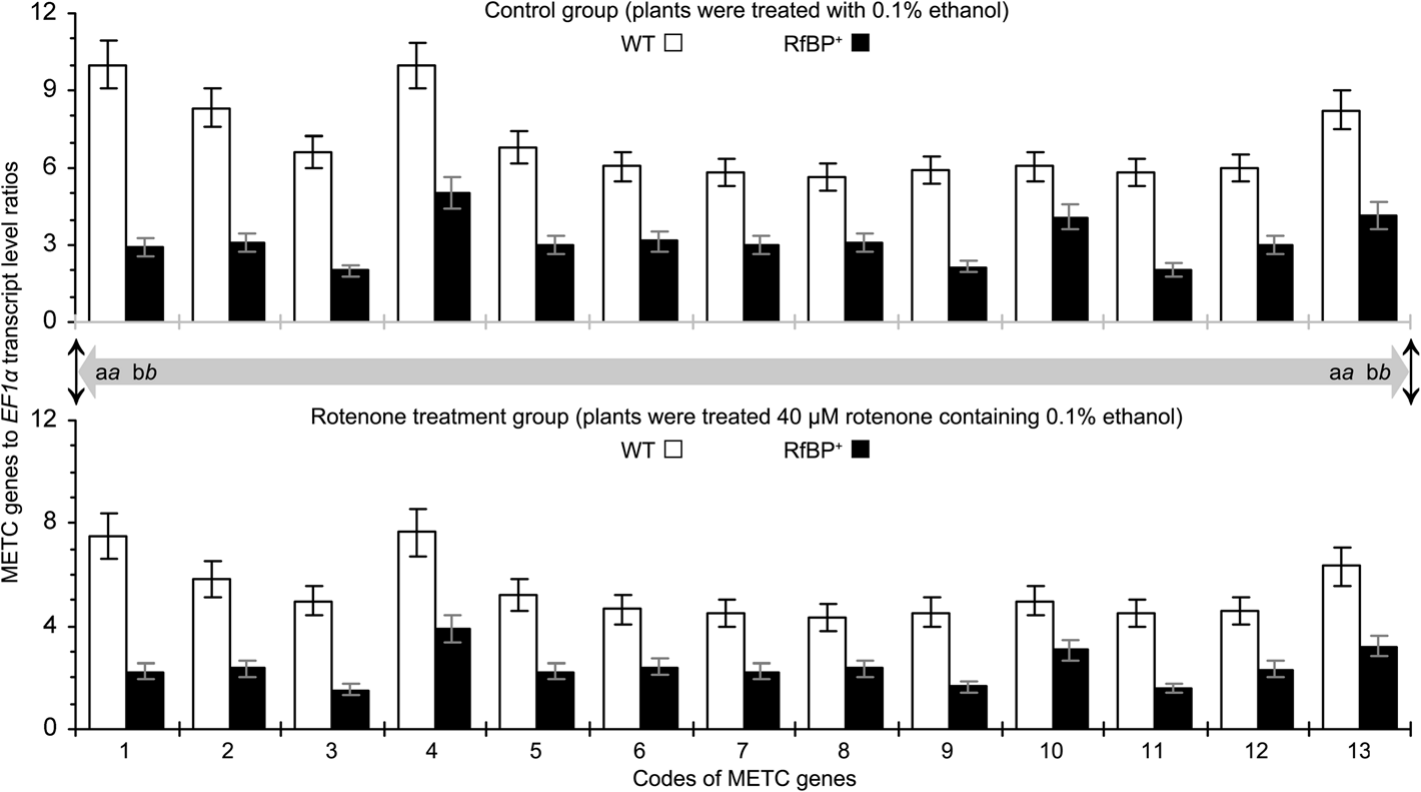
**Relative levels of METC gene expression in rotenone-treated and control plants.** Ten-day-old plants were treated with an aqueous solution containing 40 μM rotenone and 0.1% ethanol or treated with 0.1% ethanol in control. Two days later, gene expression in the two youngest expanded leaves was analyzed by real-time RT-PCR using *EF1α* as a reference gene. Data shown are average values ± standard deviations of results from six experimental repeats each containing 15 plants. Different letters in regular and *italic* fonts indicate significant differences by analysis of variance using Fisher’s least significant difference test and Tukey-Kramer’s test, respectively (n = 6; *P* < 0.01), for every of 13 data pairs shown in both bar graph panels and within the range indicated by bidirectional arrowhead grey line.

This notion was supported indirectly by analyses of METC gene expression and H_2_O_2_ concentrations in plants treated with rotenone, a ketonic chemical that inhibits electron-carrier protein complex I and induces electron leakage from this complex [18,19]. Rotenone was dissolved in ethanol and used as a water-diluted solution containing 0.1% ethanol to treat plants, and plants were treated with 0.1% ethanol in the experimental control group. Equivalent quantities of the 13 transcripts were detected in rotenone-treated and control plants irrespectively of genotype, WT or RfBP^+^ (Figure 6; Additional file 2: Figure S2). In RfBP^+^, however, rotenone treatment further reduced gene expression levels on the basis of RfBP-caused repression (Figure 6). This analysis indicated that rotenone and RfBP had a similar effect on the expression of METC genes. In contrast to the inhibitory effect on METC gene expression, rotenone treatment increased H_2_O_2_ concentrations in all plants (Figure 6). H_2_O_2_ concentrations in rotenone-treated WT and RfBP^−^ plants were elevated approximately to 90% of that in water-treated RfBP^+^ plants, indicating the similar function of rotenone and RfBP. Moreover, rotenone appeared to synergize the role of RfBP in increasing H_2_O_2_ concentrations as H_2_O_2_ in RfBP^+^ was near 50% increased by rotenone compared to control. The similar effects of rotenone and RfBP on METC gene expression and H_2_O_2_ concentrations (Figures 3, 4, 5 and 6) suggest that RfBP induces H_2_O_2_ generation possibly through electron leakage.

### RfBP-induced H_2_O_2_ contributes to early flowering

**Figure 7:**
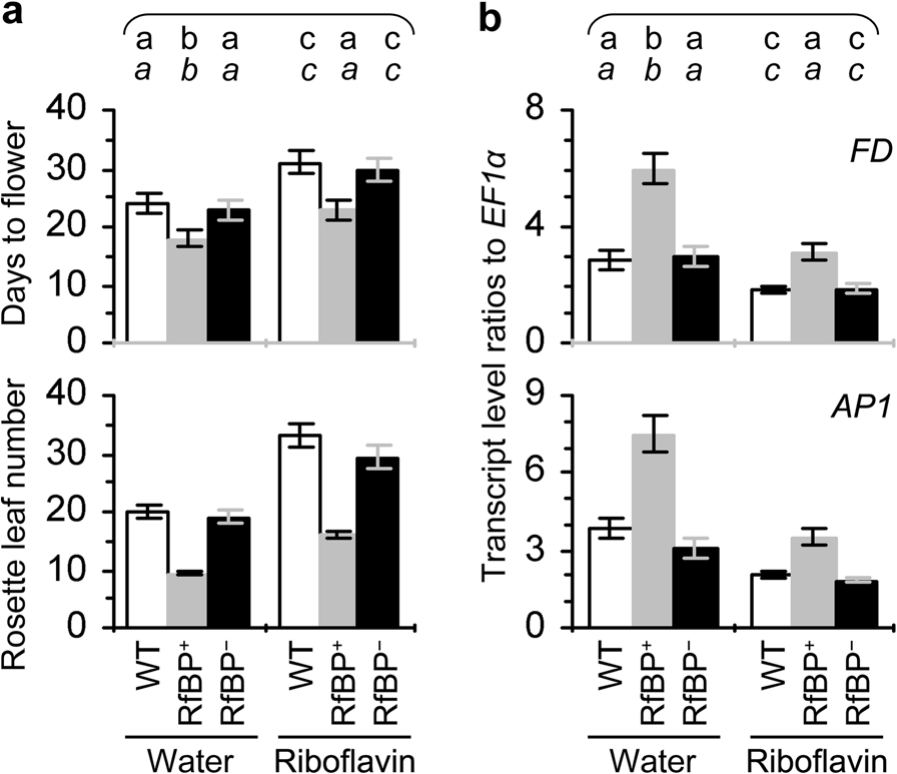
**The effects of riboflavin feeding treatment on flowering and expression of** ***FD*** **and** ***AP1*** **.** An aqueous solution of riboflavin or water was used to immerse seeds and treat 10-day-old plants by spraying over plant tops. In **(a)**, flowering time and rosette leaf number were scored. In **(b)**, relative levels of *FD* and *AP1* expression in shoot apices of 12-day-old plants were quantified by real-time RT-PCR using the constitutively expressed *EF1α* gene as a reference. Data shown are average values ± standard deviations based on three experimental repeats each containing 15 plants. On bar graphs, different letters in regular and *italic* fonts indicate significant differences by analysis of variance using Fisher’s least significant difference test and Tukey-Kramer’s test, respectively (n = 3; *P* < 0.01).

All plants flowered later with more rosette leaves when H_2_O_2_ concentrations were decreased by riboflavin feeding treatment compared to treatment with water in control (Figure 7a). The delayed flowering phenotype was coincident with decreased expression of the *FD* and *AP1* genes in shoot apices of plants fed with riboflavin (Figure 7b), which increases expression levels of METC genes in all plants and especially eliminate the inhibitory effect of RfBP on METC gene expression in RfBP^+^ (Figure 4). In RfBP^+^, riboflavin feeding treatment retrieved leaf flavins (Figure 5a), the expression of METC genes (Figure 4), *FD* and *AP1* genes (Figure 7b) to approximations of WT levels, and decreased H_2_O_2_ concentrations but did not fully cancel the RfBP-induced quantity (Figure 5c). In this case, RfBP^+^ no longer displayed the early flowering phenotype; instead, they flowered approximately as WT or RfBP^−^ plants (Figure 7a). These analyses indicate that RfBP-induced H_2_O_2_ contributes to the early flowering phenotype in correlation with enhanced expression of *FD* and *AP1* genes in the shoot apex.

### The extrinsic application of H_2_O_2_ promotes flowering

**Figure 8:**
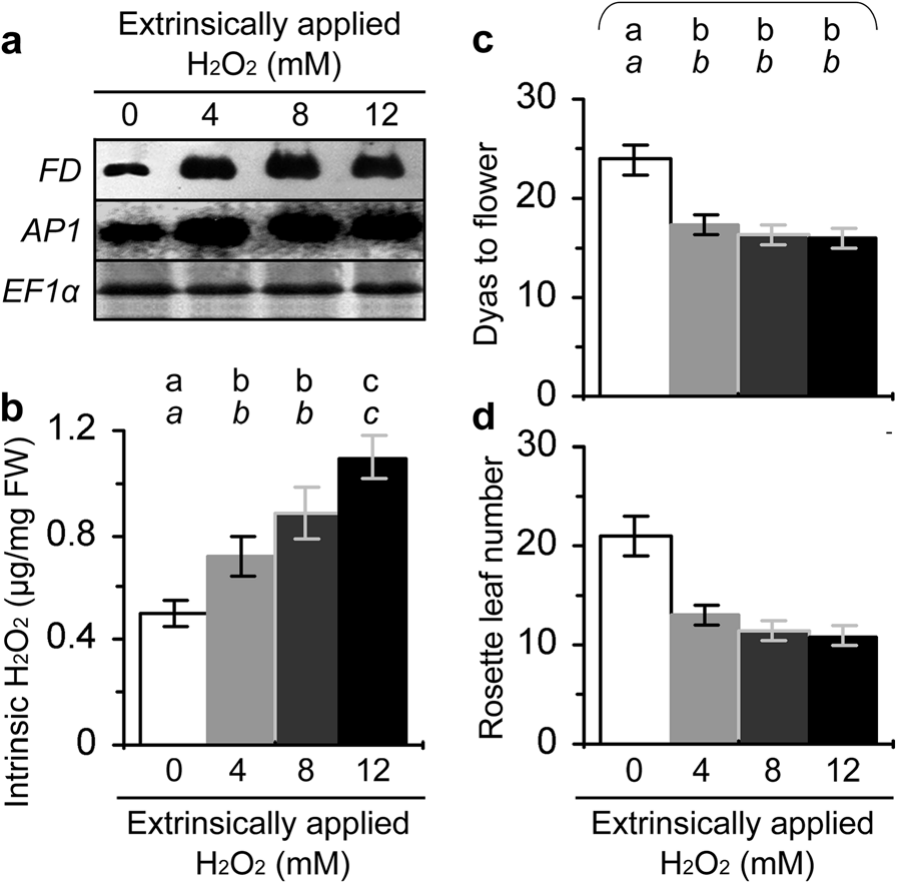
**The effects of plant treatment with H**_**2**_ **O**_**2**_ **on flowering and related responses.** Aqueous solutions of H_2_O_2_ at the indicated concentrations were used separately to immerse seeds of wild-type plants and treat seven-day-old plants grown on a medium by adding every H_2_O_2_ solution into the medium in correspondingly labeled bottles. Two days later, *FD* and *AP1* expression at the shoot apex was analyzed by Northern blotting analyses using *EF1α* gene as a reference **(a)**; H_2_O_2_ concentrations were measured by spectrometry **(b)**. Subsequently, flowering time **(c)** and rosette leaf number **(d)** were scored. Quantitative data shown are average values ± standard deviations based on three experimental repeats each containing 30 plants. On bar graphs, different letters in regular and *italic* fonts indicate significant differences by analysis of variance using Fisher’s least significant difference test and Tukey-Kramer’s test, respectively (n = 3; *P* < 0.01).

**Figure 9:**
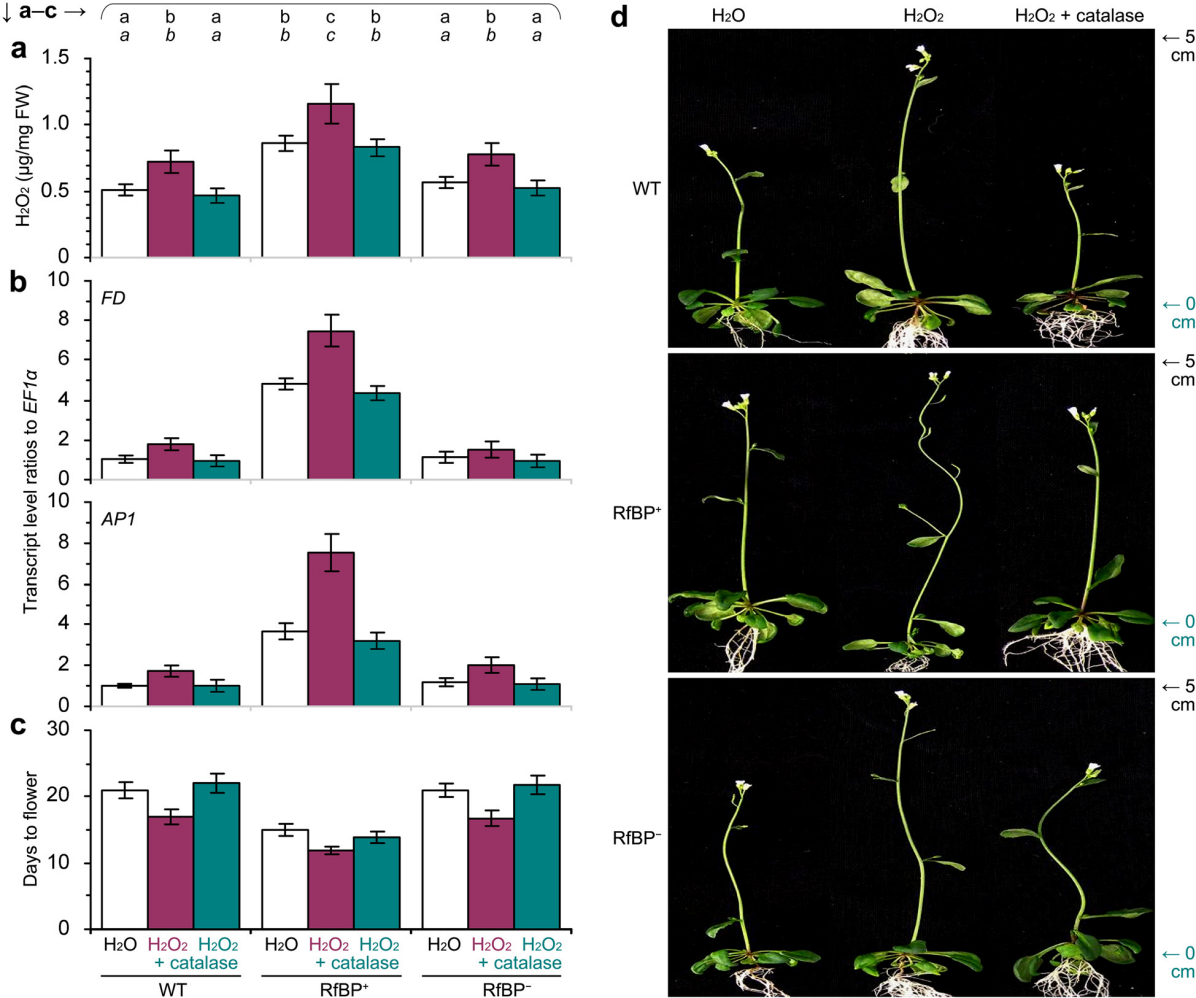
**The effects of plant treatment with H**_**2**_ **O**_**2**_ **or both H**_**2**_ **O**_**2**_ **and catalase on flowering and related responses.** Water and aqueous solutions of the indicated compounds were used separately to immerse seeds of the indicated plants and treat seven-day-old plants grown on a medium by adding every H_2_O_2_ solution into the medium in correspondingly labeled bottles. Two days later, H_2_O_2_ concentrations were measured by spectrometry **(a)**, and the expression of *FD* and *AP1* at the shoot apex was analyzed by real-time RT-PCR using the *EF1α* as a reference gene **(b)**. Flowering time was scored **(c)**, and plants were photographed after four weeks of growth **(d)**. Quantitative data shown are average values ± standard deviations based on three experimental repeats each containing 50 plants. On bar graphs, different letters in regular and *italic* fonts indicate significant differences by analysis of variance using Fisher’s least significant difference test and Tukey-Kramer’s test, respectively (n = 3; *P* < 0.01).

To confirm the promoting effect of H_2_O_2_ on flowering, we performed pharmacological studies in which plants were treated with H_2_O_2_ only or in combination with H_2_O_2_ scavenger catalase. Both compounds were used in aqueous solutions to immerse seeds and treat 10-day-old plants grown on agar medium. We first treated WT seeds and plants with a range of H_2_O_2_ concentrations and two days later we found that 4 mM H_2_O_2_ well enhanced the expression of *FD* and *AP1* in shoot apices (Figure 8a) and increased the intrinsic level of H_2_O_2_ in leaves (Figure 8b). We further found that 4 mM H_2_O_2_ was effective to induce early flowering and reduce rosette leaf number (Figure 8c,d). However, H_2_O_2_ treatment did not cause evident changes in expression levels of METC genes (Additional file 3: Figure S4). Then, we treated seeds and plants with water in control and with 4 mM H_2_O_2_ or a mixture of 4 mM H_2_O_2_ plus5 U/ml catalase. We found significant (*P* < 0.01) increases in the intrinsic H_2_O_2_ content (Figure 9a) and enhancements of *FD* and *AP1* expression (Figure 9b), and we also observed the early flowering phenotype (Figure 9c,d), in all plants treated with H_2_O_2_ compared to water. However, these effects were removed by the presence of catalase in the H_2_O_2_ treatment (Figure 9a-d). Thus, the extrinsically applied H_2_O_2_ caused a promoting effect on flowering. More precocious flowering and greater increases in the intrinsic H_2_O_2_ and in *FD* and *AP1* expression levels were observed in RfBP^+^ compared to WT and RfBP^−^ plants under the same treatment conditions (Figure 9a-d). Presumably, the extrinsic (artificially applied) and intrinsic (RfBP-induced) H_2_O_2_ cooperates to promote flowering and enhance *FD* and *AP1* expression at the shoot apex.

### H_2_O_2_ from different sources contributes to the similar effect on flowering

To elucidate whether H_2_O_2_ from different cellular sources contributes to the similar effect on flowering, we determined H_2_O_2_ concentrations, *FD* and *AP1* gene expression, and flowering time of Arabidopsis *cat2* and *nfxl1* mutants in comparison with WT and RfBP^+^ plants. Due to a mutation in peroxisomal enzyme catalase 2 (Cat2), the *cat2* mutant loses 80% of catalase activity and produces a higher level of the cytosolic H_2_O_2_ compared to the WT plant [48,49]. This was confirmed in this study by measuring leaf H_2_O_2_ concentrations, being 43% higher in *cat2* (536 ng/mg fresh leaves) than in WT (942 ng/mg) [Additional file 4: Figure S5a]. Compared to WT, *cat2* displayed higher levels of *FD* and *AP1* expression and was 10 days earlier to flower (Additional file 4: Figure S5b,c). As an indirect result of disruption in the transcription factor NFXL1, the *nfxl1* mutant incurs a 20% decrease of the cytosolic H_2_O_2_ in relative to the steady-state level [50]. Compared to WT, *nfxl1* had lower levels of cytosolic H_2_O_2_ and *FD* and *AP1* expression and displayed the late flowering phenotype leaves (Additional file 4: Figure S5b,c). These analyses suggest that H_2_O_2_ from the different sources, Cat2 or NFXL1 defection and RfBP as well, functions similarly to affect flowering time and the expression of *FD* and *AP1*.

### RfBP compensates for flowering repression in the *nfxl1* mutant

**Figure 10:**
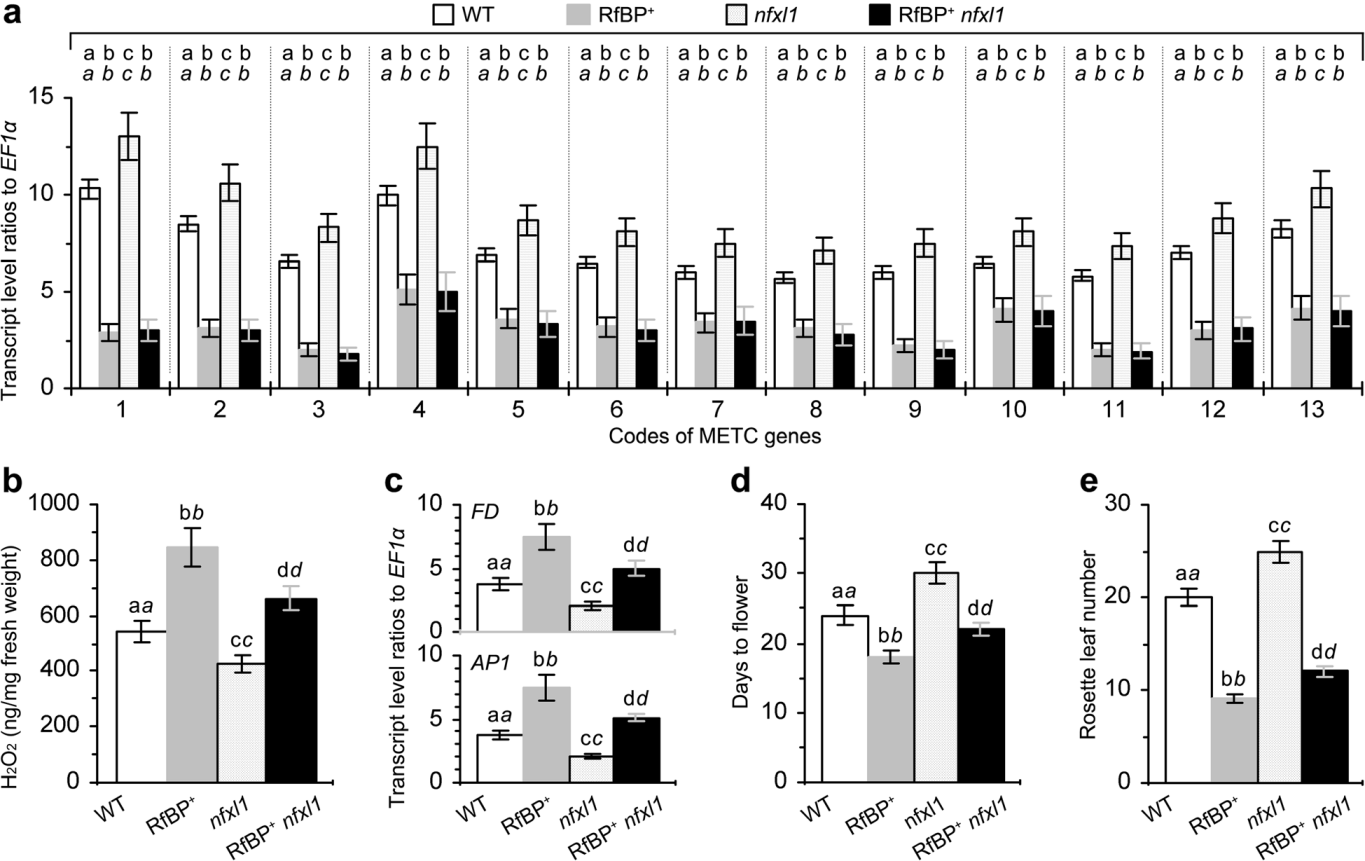
**The antagonistic effects of RfBP and** ***NFXL1*** **gene deletion (** ***nfxl1*** **) on flowering and related responses.** Shown here are measurements on METC gene expression in leaves **(a)**, leaf H_2_O_2_ concentrations **(b)**, *FD* and *AP1* expression at the shoot apex **(c)**, flowering time **(d)**, rosette leaf number **(e)**. The antagonistic effects were analyzed on WT, RfBP^+^, *nfxl1* mutant, and RfBP^+^ *nfxl1* hybrid plants. Gene expression was quantified by real-time RT-PCR using *EF1α* as a reference gene and H_2_O_2_ was measured by spectrometry, and both analyses were performed on 12-day-old plants. Data shown are mean values ± standard deviations based on three experimental repeats each containing 30 plants. On bar graphs, different letters in regular and *italic* fonts indicate significant differences by analysis of variance using Fisher’s least significant difference test and Tukey-Kramer’s test, respectively (n = 3; *P* < 0.01).

Because *RfBP*^*+*^ and *nfxl1* are opposite and likely to counteract the role in H_2_O_2_ content alterations and the effect on flowering, both plants were crossed and the RfBP^+^*nfxl1* hybrid was generated for further analyses. METC genes were expressed similarly in RfBP^+^*nfxl1* and RfBP^+^ plants (Figure 10a), suggesting that the *nfxl1* mutation was unrelated to METC gene expression. However, the hybrid appeared to be intermediate of both parents in the cytosolic H_2_O_2_ content (Figure 10b), levels of *FD* and *AP1* expression (Figure 10c), flowering time (Figure 10d), and rosette leaf number (Figure 10e). Clearly, RfBP^+^ compensates for flowering repression in the *nfxl1* mutant.

## Discussion

This study was attempted to mainly elucidate how H_2_O_2_ is induced by RfBP to affect flowering time on the basis of our recent evidence that early flowering is a constant phenotype conferred by *de novo* expression of the turtle *RfBP* gene and associated with a constant increase of leaf H_2_O_2_ concentrations and timely enhanced expression of *FD* and *AP1* at the shoot apex in RfBP^+^ Arabidopsis plants under short days, long days, or inductive photoperiod [40]. Under these conditions, enhanced expression of *FD* and *AP1* is essential for floral organ formation at the shoot apex [38,39,52] and well reflects the molecular basis of RfBP-induced early flowering [40]. In this study, we simplified the experiment system by growing plants only in long days and under this condition we correlated the early flowering phenotype with enhanced expression of *FD* and *AP1*, floral regulatory and FMI genes, respectively (Figure 1). Data obtained from multiple experimental repetitions demonstrated that: (i) RfBP represses the expression of 13 of 19 METC genes (Figures 2, 3 and 4; Additional file 1: Figures S1 and Additional file 2: Figure S2) and induces H_2_O_2_ probably results from electron leakage at METC (Figures 5 and 6; Additional file 3: Figure S4 and Additional file 5: Figure S3); (ii) H_2_O_2_ promotes flowering and enhances the expression of *FD* and *AP1* (Figures 7, 8 and 9); and (iii) the potential electron leakage appears to be one of biochemical sources for the generation of H_2_O_2_ with the promoting effect on flowering (Figure 10; Additional file 4: Figure S5). Previously we showed that the foreign RfBP protein is capable of modulating the intrinsic content of free flavins with physiological and pathological consequences. Inside the RfBP^+^ cell, RfBP binds with riboflavin, reduces quantities of free flavins in leaves, and concomitantly elevates concentrations of the cytosolic H_2_O_2_, which acts in turn to regulate defense responses to a bacterial pathogen [13]. Therefore, flavin content downregulation by the foreign RfBP protein has developmental and defensive consequences.

In recent 10 years, genetic modification of the riboflavin biosynthesis pathway alters some aspects of plant development, such as leaf senescence regulated by the COS1 protein characteristic of lumazine synthase, which catalyzes the penultimate step of the riboflavin biosynthesis pathway [53] and is an essential component of jasmonic acid signaling pathway [54]. In plants, moreover, externally applied riboflavin induces plant growth enhancement by activating ethylene signaling pathway [55]. Externally applied riboflavin also induces resistance to pathogens in a manner of salicylic acid dependence or independence according to the type of pathogens [26,41]. These findings suggest that changes in riboflavin content cause physiological and pathological responses by affecting phytohormone signaling pathways. Based on our studies detailed here and reported earlier [40], novel functions of flavins have been extended from hormone signaling to flowering time control.

Early flowering associates with spontaneously repressed expression of 13 of 19 METC genes (Figures 2, 3 and 4; Additional file 1: Figure S1 and Additional file 2: Figure S2) and concomitantly elevated cytosolic H_2_O_2_ concentrations (Figure 5) in RfBP^+^ plants. The repression on METC genes is attributable to decreased concentrations of free flavins in leaves and can be removed either by RfBP^−^ or by riboflavin feeding treatment under RfBP^+^ background (Figures 3 and 4; Additional file 1: Figure S1 and Additional file 2: Figure S2). Based on the roles of RfBP^−^ and riboflavin feeding in restoring RfBP^+^ to WT in flavin content and flowering time, as well as different extents by which riboflavin feeding and RfBP increase H_2_O_2_ concentrations (Figures 5 and 7), increased H_2_O_2_ is at least partially caused by RfBP-reduced flavin content and contributes to the early flowering phenotype. On one hand, direct evidence for the promoting effect of H_2_O_2_ on flowering was found in the pharmacological analysis with H_2_O_2_ and catalase (Figures 8 and 9). On the other hand, the coincident decreases in levels of three flavins (Figure 5) conform to dynamics of flavin form conversions. Riboflavin and FMN conversion is reversible [56], while FMN to FAD conversion is irreversible [57]. Accumulation of a particular flavin is concentration-dependent, so that a smaller amount of free riboflavin or FMN results in a smaller amount of FMN or FAD [1]. Therefore, downregulation of free riboflavin is an initial cause of coordinate decreases of free FMN and FAD concentrations and is also a cause of the subsequent effect on flowering time under RfBP^+^ background. In RfBP^+^, however, although riboflavin feeding treatment only increases H_2_O_2_ but does not retrieve it to the WT level (Figure 5), the treatment enables RfBP^+^ to resemble WT in flavin content, the expression of METC, *FAD*, and *AP1* genes, and flowering time in particular (Figures 4 and 7). This discrepancy indicates that downregulating flavin content is not the only mechanism by which RfBP induces H_2_O_2_ and early flowering. Alternatively, the extrinsically applied riboflavin is insufficiently effective as the intrinsically produced flavins to affect cellular redox. At this point, we are unable to pertinently prospect the relationship between flavin-mediated redox and flowering time control based on the riboflavin-feeding experiment.

Regarding to the riboflavin-feeding effect, a question is how the extrinsically applied riboflavin increases the intrinsic flavin concentrations. In plants, riboflavin synthesis, its conversion to FMN, and FMN conversion to FAD are predicted to occur in plastids [56,57]. With the cell growth, plastids differentiate into chloroplasts [58], in which RfBP is localized [13]. Flavins are transported by subcellular trafficking and function in processes such as METC [59]. In animals, RfBP functions to mediate the cellular translocation of riboflavin [60,61]. Animals absorb riboflavin directly from dietary sources [62] or produce this vitamin through conversions from ingested FMN and FAD [63]. In both cases, RfBP acts to redistribute riboflavin between cellular compartments, between cells, and from one organ to another [30,60]. A similar trafficking mechanism may be responsible for transport of the extrinsic riboflavin into plant cells but this hypothesis remains to be examined.

Regarding to the effects of RfBP on flavin levels and METC gene expression, an important question is how RfBP-decreased flavin concentrations cause H_2_O_2_ generation potentially through electron leakage. Because electron-carrier protein complexes I and II involve the first five of RfBP-repressed 13 genes, and employ FMN/FMNH_2_ and FAD/FADH_2_ as redox centers (Figure 2), respectively, electrons may leak from both complexes due to RfBP-reduced flavin concentrations, resulting in increased concentrations of the cytosolic H_2_O_2_ (Figure 5). This postulation was indirectly supported by circumstantial evidence as following: (i) similar dual roles that rotenone and shortage of flavins play in repressing METC gene expression and increasing H_2_O_2_ (Figures 4, 5 and 6; Additional file 5: Figure S3); (ii) the ternary effects of riboflavin feeding in increasing the intrinsic flavin and METC gene expression levels but decreasing H_2_O_2_ concentrations (Figures 4, 5 and 7); and (iii) the lack of effect of the externally applied H_2_O_2_ on METC gene expression (Figure 6; Additional file 3: Figure S4). In addition, a key point in (i) and (ii) is that the increase of H_2_O_2_ concentrations is a result, but not a cause, of repressed METC gene expression. These analyses suggest the possibility that flavin shortage due to downregulation by RfBP causes the repressive effect as does the toxicity of rotenone [21] on METC to induce electron leakage.

Mechanisms by which rotenone and RfBP repress METC gene expression may be different according to natures of rotenone RfBP, as well as components and functions of METC. Rotenone is a broad-spectrum insecticide, pesticide, and piscicide, is toxic to METC, impairs the role of electron carrier-protein complex I [64] in transport of electron tetrad to single O_2_ accepter, and inhibits O_2_ reduction to form H_2_O with protons from NADH_2_ and FADH_2_ [15-17]. As regards the effect of RfBP, RfBP-caused shortage of FMN and FAD [13] may lead to insufficient functions of FMN to receive protons from NADPH in complex I and FAD to supply protons for CoQ in complex II (Figure 2). Electron leakage and H_2_O_2_ generation subsequent to complex I inhibition by rotenone have been well studied in animals [20,21,64], but little was known about whether plants incur a similar inhibition. Owing to the alternative oxidization bypass located between complex III and the inner membrane-associated CoQ, inhibition by rotenone may not cause electron leakage from complex III, but electrons are still likely to leak from complex I or II [15,16,63]. Moreover, inhibition of plant complex I expands impacts far beyond the complex itself since a number of metabolic pathways associated with mitochondria and other organelles are altered concomitantly [65]. This also explains a possible way, alternative to reducing flavin content, by which RfBP affects H_2_O_2_ accumulation and flowering time. Repressing METC gene expression seems to be a mode of the rotenone action on METC so that the consequence is similar in rotenone-treated WT and water-treated RfBP^+^ plants (Figure 6; Additional file 2: Figure S2). At present, however, there is no evidence to elucidate the mechanism by which rotenone causes repression of METC gene expression.

In addition to electron leakage from METC, H_2_O_2_ can be generated by many other mechanisms at different sites in plant cells through flavin-mediated cytosolic and peroxisomal redox processes [66]. Cytosolic and peroxisomal redox genes whose expression levels are more than 50% reduced by RfBP encode single alutaredoxin (At1G03850), thioredoxin (At1G07960), and peroxiredoxin type 2 (At1G60740) proteins and three glutaredoxin (At1G77370, At5G18600, and At5G40370) proteins (http://www.ncbi.nlm.nih.gov/geo/query/acc.cgi?acc=GSE18417). All these proteins are H_2_O_2_ scavengers and may function concomitantly with the potential electron leakage mechanism to elevate cytosolic concentrations of H_2_O_2_ and affect flowering time. As stated above, therefore, the repressive effect on METC gene expression is not the only mechanism by which RfBP induces H_2_O_2_ and promotes flowering under RfBP^+^ background. Testing of this hypothesis obviously needs numerous challenged genetic, biochemical, and molecular studies. In the present study, we are only able to provide indirect evidence that RfBP^+^ and *nfxl1* plants mutually counteract in altering cytosolic H_2_O_2_ concentrations and in the subsequent effects on flowering time and expression of *FD* and *AP1* at the shoot apex (Figure 10; Additional file 4: Figure S5).

With respect to H_2_O_2_ generation and function at different sites in plant cells, a critical question is related to cellular translocation of H_2_O_2_, or how mitochondrial H_2_O_2_ is recruited into flowering time control? Compared to the apoplastic-cytoplasmic translocation [13], intracellular translocation of H_2_O_2_ subsequent to generation in different cellular compartments may play more important roles in regulating multiple physiological processes [11]. The supposed H_2_O_2_ translocation may not depend on free diffusion, but instead, it may comply with certain modes of the selectivity [67,68]. In a great attention, the aquaporin channel originally assigned to water transport [69-71] has been implicated in cellular translocation of other small compounds [72,73] including H_2_O_2_ [67,74]. Particular aquaporins may mediate H_2_O_2_ translocation for its recruitment into flowering time control. This hypothesis needs to be tested.

## Conclusions

We have shown that RfBP-induced H_2_O_2_ presumably results from METC electron leakage due to flavin downregulation by RfBP, H_2_O_2_ is a positive regulator of flowering, and the hypothetical electron leakage appears to be one of biochemical sources of H_2_O_2_ with the promoting effect on flowering. In fact, early flowering is a serendipitous phenomenon associated with the *de novo* expression of *RfBP*, but we don’t exactly know what it means with respect to flavin-mediated redox and flowering time control.

## Methods

### Plant material and growth conditions

The *RfBP*-expressing *Arabidopsis thaliana* line was previously designated as REAT11 [13] and recently renamed RfBP^+^ [40]. The *RfBP*-silenced line RfBPi11 generated under RfBP^+^ (REAT11) background [13] was renamed RfBP^−^ [40]. The *cat2* and *nfxl1* mutants were generated previously by T-DNA insertion into the *Cat2* gene [48,49] and the *NFXl1* gene [50]. Their seeds were purchased from The Arabidopsis Information Resource (http://www.arabidopsis.org) under stock numbers SALK_076998 and SALK_001399, respectively. Seeds of other plants were maintained in this lab. Plants were grown in pots containing potting soil [75] or on Murashigie and Skoog (MS) medium under environment-controlled conditions: 22 ± 1°C, 55% ± 2% humidity, long days (16-hour light and 8-hour dark), and light at 200 μM quanta/m^2^/s. Day 0 was considered after stratification. The flowering phenotype was characterized by rosette leaf number and days to flower scored with 50 plants of every genotype in each of seven independent experimental repeats.

### Gene expression analysis

Total RNA was isolated from combined samples of the two youngest expanded leaves excised directly or from shoot apices, which were excised under a binocular microscope, from 15 plants in every of three or six experimental repeats. Isolated RNA was subjected to Northern (RNA) blotting or quantitative real-time reverse transcriptase-polymerase chain reaction (RT-PCR) analyses using the constitutively expressed *EF1α* gene as a reference. Northern blots were hybridized to the *RfBP*-specific probe labeled with digoxigenin (EMD Biosci. Inc., Madison, WI, USA). Real-time RT-PCR was performed with specific primers (Additional file 6: Table S1) and followed previously described methods [76,77]. Genes were amplified <26 cycles with a range of template concentration increases by 0.5 ng and from 0 to 3.0 ng in 25 μl reaction solutions to select desired doses. The 25 μl reaction mixture was composed of 1 μl first-strand cDNA diluted 1:10, 2.5 μM primer and 1 × SYBR Premix Ex Taq (TaKaRa Biotech. Co., Ltd, Dalian, China). In each of three experimental repeats, all reactions were performed in triplicate with null-template controls in which cDNA was absent. Relative expression level of a tested gene was quantified as the ratio of transcript amounts between the tested gene and *EF1α*. Relative expression levels were shown directly or converted to percentages for pharmacological treatments vs. control (treatment with water) or for RfBP^+^ and RfBP^−^ plants compared to WT.

### Protein analysis

A histidine (His) tag had been added to the C-terminus of RfBP in the transformation construction and was used to facilitate purification of plant proteins by nickel chromatography [13,76]. The two youngest expanded leaves were excised and used in isolation of total proteins from 10 mg fresh leaves as previously described [78]. Isolated proteins were bound to nickel-polystyrene beads according to the manufacturer’s instruction (Amersham Biosciences Corp., Piscataway, NJ, USA), eluted with aqueous solutions of imidazole at 100, 150, and 300 mM, respectively. The 200-mM imidazole eluent was treated with the Novagen Enterokinase Cleavage Capture Kit (EMD Biosciences Inc., Darmstadt, Germany) to remove the His tag and analyzed by tricine sodium dodecyl sulfate polyacrylamide gel electrophoresis [76]. Proteins were visualized by gel staining with Coomassie G-250.

### Pharmacological study

The riboflavin feeding experiment was performed on plants grown in pots. Riboflavin (EMD Biosci. Inc., Darmstadt, Germany) was prepared as a 0.1 mM aqueous solution, amended with 0.03% (v/v) Silwet-L77 as a surfactant, and applied to 10-day-old plants by spraying plant tops with an atomizer [79]. Plants were treated similarly with an aqueous solution containing 0.03% Silwet-L77 in the experimental control group. Two days later, shoot apices were excised as stated above and used in the analysis of *FD* and *AP1* gene expression, and the two youngest expanded leaves were excised and used to detect the subcellular distribution and concentrations of H_2_O_2_, as well as expression of METC genes.

The effects of rotenone (Sigma-Aldrich, St. Luis, WA, USA) on METC gene expression and H_2_O_2_ concentrations were analyzed by experiments as for the riboflavin feeding experiment. Rotenone was prepared as a 40 mM solution in 100% (v/v) ethanol, diluted with water, and used at 40 μM in an aqueous solution containing 0.1% ethanol to treat 10-day-old plants by spraying over plant tops. Plants were treated similarly with 0.1% ethanol in control.

The effects of H_2_O_2_ and catalase (Sigma-Aldrich) on the intrinsic H_2_O_2_ concentrations and flowering time were determined using plants grown on MS medium in glass bottles (5 cm high, 2 and 1.5 cm wide diametrically at top and bottom). Aqueous solutions of 4, 8, and 12 mM H_2_O_2_ and 5 μU/ml catalase were used after sterilization with 0.22 μm cellulose filters. Seeds were sterilized and subjected to two types of experiments. The first type was devised to estimate functional dosage of H_2_O_2_ in a range of 0 (sterile water only), 4, 8, and 12 mM applied in separate seed immersion. The second type of experiment was to test the combinative effect of H_2_O_2_ and catalase. Sterilized seeds were immersed with 4 mM H_2_O_2_ or 5 U/ml catalase or their mixture and immersed with sterile water in the experimental control group. Seed immersion was maintained six hours under room temperature and then washed with sterile water for five times under sterile conditions. Washed seeds were sowed on the medium in sterile 300-ml plastic bottles. Ten days later (when plants were seven days old), the medium were supplied with 5 μM H_2_O_2_, 5 μU/ml catalase, or both and with sterile water in the control group. After incubation for additional two days, plants were used in the analysis of *FD* and *AP1* expression in shoot apices and flavin concentrations in the two youngest expanded leaves. Flowering time and rosette leaf number were scored.

### H_2_O_2_ detection

Subcellular localization of H_2_O_2_ was detected by fluorescent H_2_O_2_ probes Amplex Red (AR) and Amplex Ultra Red (AUR) (Invitrogen, San Diego, CA, USA) as previously described [9,13,80]. Both probes were used because previous observations showed that AR and AUR were oxidized in reaction with H_2_O_2_ to emit strong crimson fluorescence [9,81]. The two youngest expanded leaves were excised and immediately immersed in the pH7.4 phosphate buffer solution containing 10 μM AR or AUR, and were incubated within the solution in dark for 3 hours under a low pressure provided by a vacuum pump and a bell jar. Probed samples were observed under the ZEISS LSM700 laser scanning confocal microscope. The fluorescence emission of oxidized AR and AUR was observed between 585 and 610 nm using 543-nm argon laser excitation.

The content of H_2_O_2_ in plants was determined by quantifying the leaf H_2_O_2_ extract with a spectrophotometer. H_2_O_2_ was extracted from the first and second youngest leaves of 15-day-old plants and quantified by monitoring A_415_ of the titanium-peroxide complex formed with the H_2_O_2_ extract [26]. The content of H_2_O_2_ in plant leaves was determined according to the A_415_ curve of the titanium-peroxide complex formed with a range of standard H_2_O_2_ from a commercial source [26].

### Generation of the RfBP^+^*nfx1* hybrid

RfBP^+^ and *nfx1* plants were crossed on 10 days after flowering by pollinating *atnfx11* pistils with RfBP^+^ microspore. RfBP^+^ carries an *IPT II* gene [13], and *nfnxl1* carries *IPT II* and a T-DNA insert (http://signal.salk.edu/tdna_protocols.html). Therefore, the RfBP^+^*nfxl1* hybrid was identified based on growth in kanamycin-containing MS medium and PCR analyses of both *RfBP* and the insertion flanking sequence (Additional file 6: Table S1). The hybrid was self-crossed and its homologous F3 progenies were used in this study.

### Data analysis

All experiments were carried out by completely randomized design and repeated at least three times with similar results. Quantitative data were analyzed with commercial IBM SPSS19.0 software package (IBM Corporation, Armonk, NY, USA; http://www-01.ibm.com/software/analytics/spss/). Homogeneity-of-variance in data was determined by Levene test, and formal distribution pattern of the data was confirmed by Kolmogorov-Smirnov test and P-P Plots [82]. Then, data were subjected to analysis of variance along with Fisher’s least significant difference test [83] and Tukey-Kramer’s test [84], respectively, using commercial SPSS19.0 software package.

### Availability of supporting data

The microarray data supporting the results of this article are available in NCBI Gene Expression Omnibus repository (http://www.ncbi.nlm.nih.gov/geo/) under accession number GSE18417.

## Additional files

Additional file 1: Figure S1. Relative levels of METC gene expression in WT, RfBP^+^, and RfBP^−^ plants. Additional file 2: Figure S2. Relative levels of METC gene expression in rotenone-treated and control plants. Additional file 3: Figure S4. Relative levels of METC gene expression in H_2_O_2_-treated and control plants. Additional file 4: Figure S5. Comparisons of RfBP^+^ and *Cat2* and *NFXL1* mutations in H_2_O_2_ concentrations, *FD* and *AP1* expression, and flowering time. Additional file 5: Figure S3. The effect of rotenone on H_2_O_2_ concentrations in leaves. Additional file 6: Table S1. Information on genes analyzed and primers used in this study.

## Abbreviations

AP1: APETALA1
AR: Amplex red
AUR: Amplex ultra red
*cat2*: an Arabidopsis mutant with T-DNA-indexed *CAT2* gene and decreased H_2_O_2_ content
CoQ: Ubiquinone
Cyt: Cytochrome
FAD: Flavin adenine dinucleotide
FMI: Floral meristem identity
FMN: Flavin mononucleotide
METC: Mitochondrial electron transport chain
NAD: Nicotinamide adenine dinucleotide
*nfxl1*: an Arabidopsis mutant with T-DNA-indexed *NFXL1* gene and decreased H_2_O_2_ content
RfBP: Riboflavin-binding protein
RfBP^+^: RfBP-expressing transgenic Arabidopsis line
RfBP^−^: RfBP-silenced Arabidopsis line generated under RfBP^+^ background
ROS: Reactive oxygen species
