# Supplementary material for: Expression of turtle riboflavin-binding protein represses mitochondrial electron transport gene expression and promotes flowering in Arabidopsis
Source: BMC Plant Biol. 2014 Dec 30;14:381. doi: 10.1186/s12870-014-0381-5 (PMC4310184; doi:10.1186/s12870-014-0381-5)
Supplement: Additional file 1: Figure S1. — Relative levels of METC gene expression in WT, RfBP+, and RfBP− plants. [file 12870_2014_381_MOESM1_ESM.pdf]

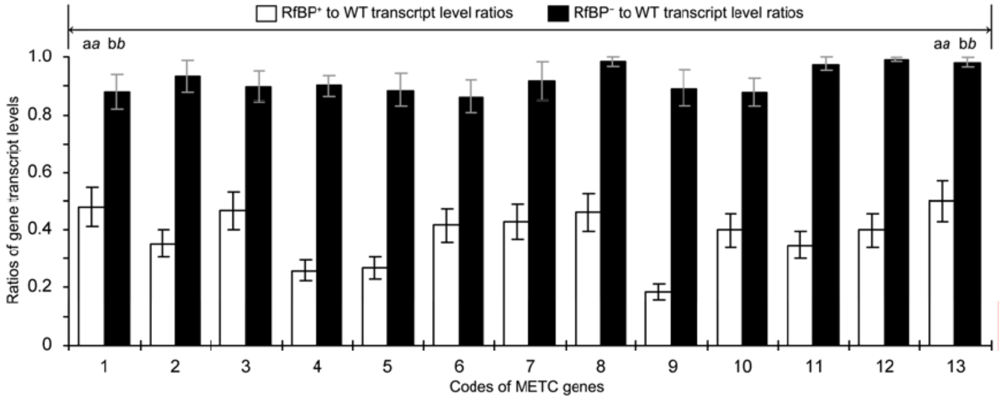

Additional file 1: Figure S1. Quantitative ratios of METC gene transcripts between $RfBP^{+}$ and WT and between $RfBP^{-}$ and WT plants.

Relative expression levels of the tested METC genes in contrast to the reference gene (Figure 3) were used in plant pair comparisons. Data shown are mean values ± standard deviations from six experimental repeats each containing 15 plants. On bar graphs, different letters in regular and *italic* fonts indicate significant differences by analysis of variance using Fisher's least significant difference test and Tukey-Kramer's test, respectively ($n = 6$; $P < 0.01$).
