# Supplementary material for: Expression of turtle riboflavin-binding protein represses mitochondrial electron transport gene expression and promotes flowering in Arabidopsis
Source: BMC Plant Biol. 2014 Dec 30;14:381. doi: 10.1186/s12870-014-0381-5 (PMC4310184; doi:10.1186/s12870-014-0381-5)
Supplement: Additional file 2: Figure S2. — Relative levels of METC gene expression in rotenone-treated and control plants. [file 12870_2014_381_MOESM2_ESM.pdf]

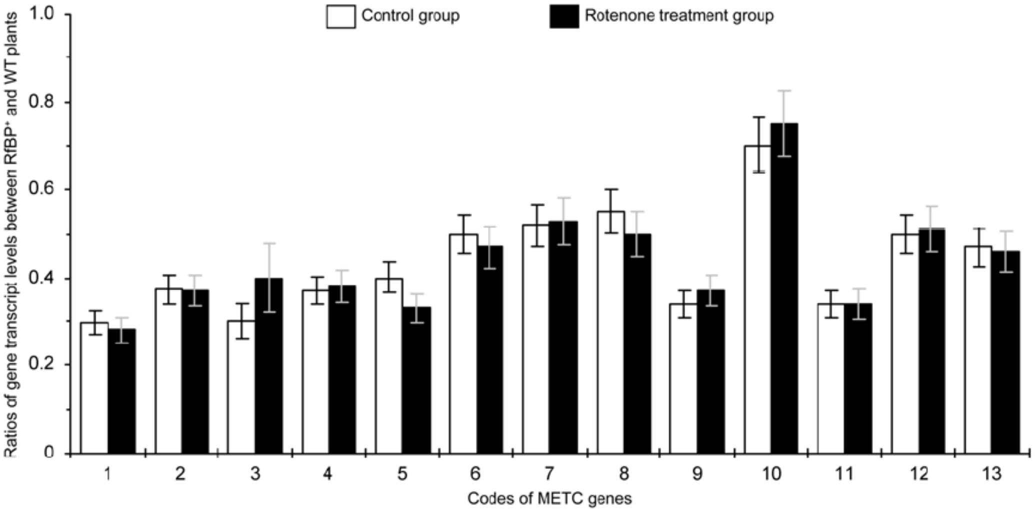

Additional file 2: Figure S2. The effect of rotenone on METC gene expression. Ten-day-old plants were treated with treated with 0.1% ethanol (control group) or an aqueous solution containing 40 μM rotenone (rotenone treatment group). Two days later, gene expression in leaves was analyzed by real-time RT-PCR using *EF1α* as a reference gene (Figure 6). Relative expression levels of METC genes were used in pair comparisons between $RfBP^+$ and WT plants. Data shown are mean values ± standard deviations from six experimental repeats each containing 15 plants.
