# Supplementary material for: Expression of turtle riboflavin-binding protein represses mitochondrial electron transport gene expression and promotes flowering in Arabidopsis
Source: BMC Plant Biol. 2014 Dec 30;14:381. doi: 10.1186/s12870-014-0381-5 (PMC4310184; doi:10.1186/s12870-014-0381-5)
Supplement: Additional file 3: Figure S4. — Relative levels of METC gene expression in H2O2-treated and control plants. [file 12870_2014_381_MOESM3_ESM.pdf]

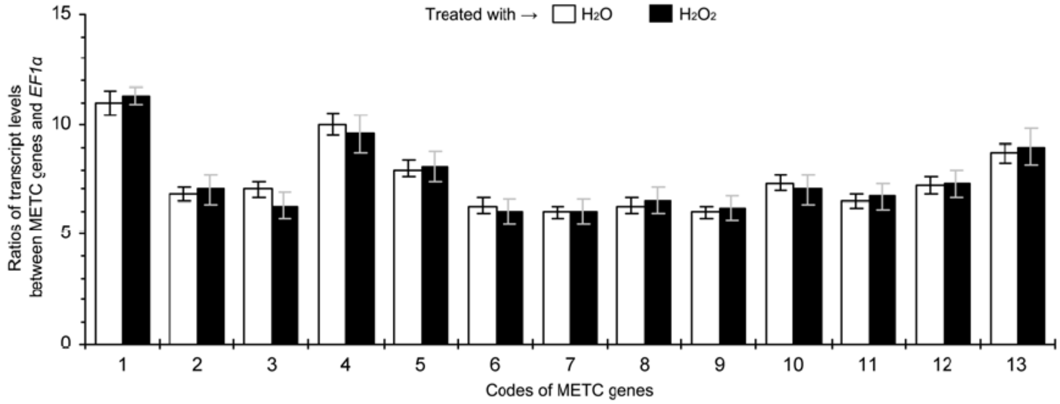

Additional file 4: Figure S4. Relative levels of METC gene expression in H_2O_2-treated and control plants.

Water or a 4 μM aqueous solution of H_2O_2 was used to immerse seeds and treat 7-day-old plants. Gene expression in leaves of 9-day-old plants was analyzed. Data shown are average values ± standard deviations of results from three experimental repeats each containing 15 plants.
