# Supplementary material for: Expression of turtle riboflavin-binding protein represses mitochondrial electron transport gene expression and promotes flowering in Arabidopsis
Source: BMC Plant Biol. 2014 Dec 30;14:381. doi: 10.1186/s12870-014-0381-5 (PMC4310184; doi:10.1186/s12870-014-0381-5)
Supplement: Additional file 4: Figure S5. — Comparisons of RfBP+ and Cat2 and NFXL1 mutations in H2O2 concentrations, FD and AP1 expression, and flowering time. [file 12870_2014_381_MOESM4_ESM.pdf]

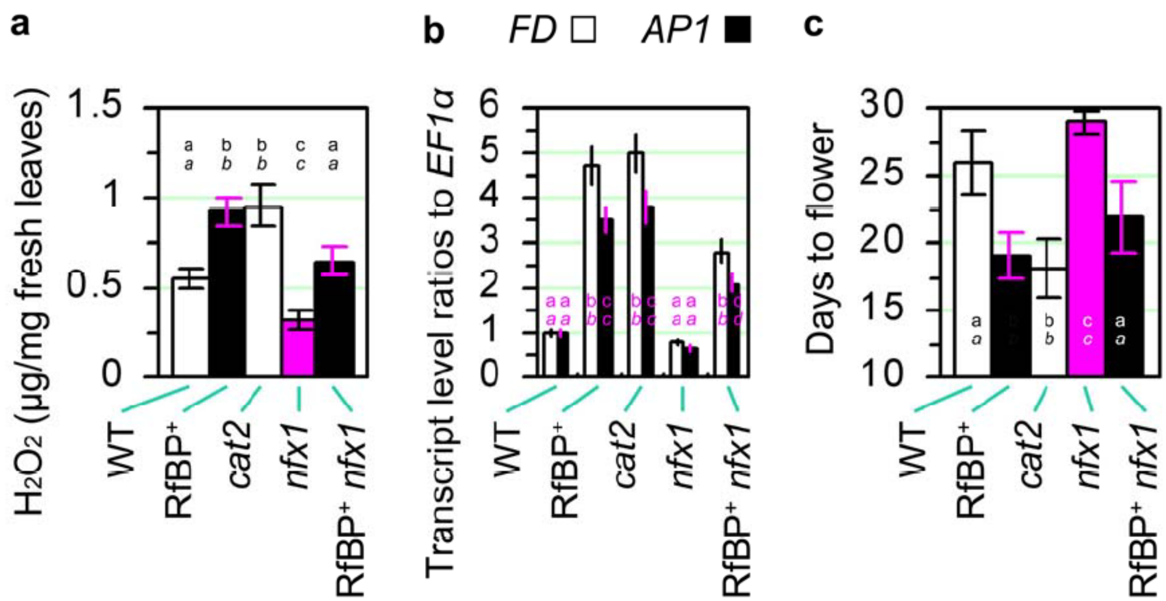

Additional file 5: Figure S5. Comparisons of RfBP^{+} and *Cat2* and *NFXL1* mutations in H_2O_2 concentrations, *FD* and *AP1* expression, and flowering time.

Shown here are H_2O_2 concentrations in leaves (**a**), *FD* and *AP1* expression at the shoot apex (**b**), and flowering time (**c**) of the different plants. H_2O_2 concentrations and gene expression were analyzed on 12-day-old plants. Data shown are average values ± standard deviations of results from three experimental repeats each containing 15 plants (**a,b**) and 50 plants (**c**). In bar graphs, different letters in regular and *italic* fonts indicate significant differences by analysis of variance using Fisher's least significant difference test and Tukey-Kramer's test, respectively ($n = 3$; $P < 0.01$).
