# Supplementary material for: Expression of turtle riboflavin-binding protein represses mitochondrial electron transport gene expression and promotes flowering in Arabidopsis
Source: BMC Plant Biol. 2014 Dec 30;14:381. doi: 10.1186/s12870-014-0381-5 (PMC4310184; doi:10.1186/s12870-014-0381-5)
Supplement: Additional file 5: Figure S3. — The effect of rotenone on H2O2 concentrations in leaves. [file 12870_2014_381_MOESM5_ESM.pdf]

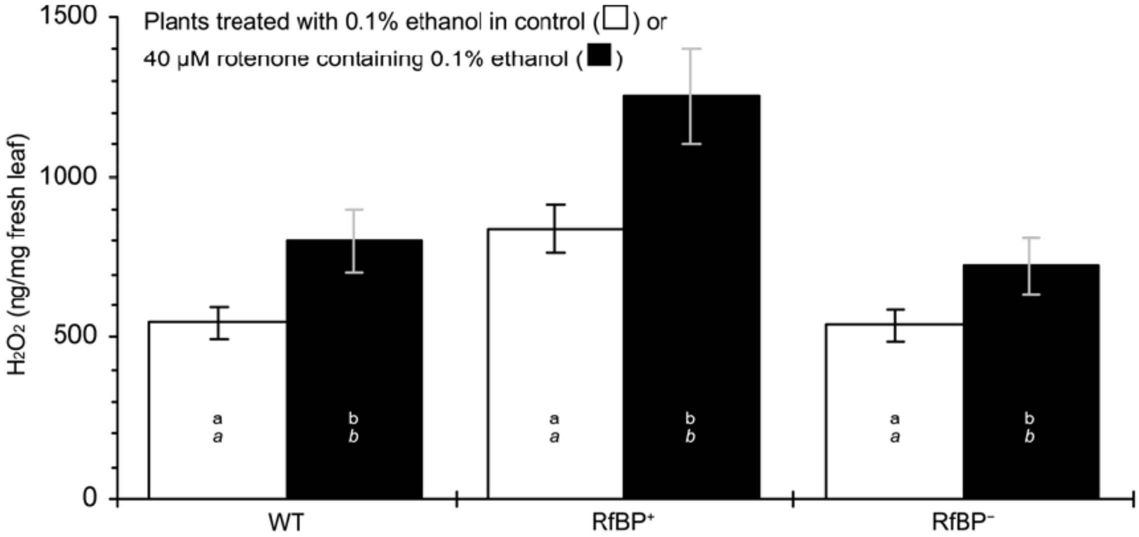

Additional file 3: Figure S3. The effect of rotenone on H_2O_2 concentrations in leaves. Ten-day-old plants were treated and two days later, H_2O_2 concentrations in leaves were measured. Data shown are average values ± standard deviations of results from three experimental repeats each containing 15 plants. In bar graphs, different letters in regular and *italic* fonts indicate significant differences by analysis of variance using Fisher's least significant difference test and Tukey-Kramer's test, respectively ($n = 3$; $P < 0.01$).
