# Supplementary material for: Expression of turtle riboflavin-binding protein represses mitochondrial electron transport gene expression and promotes flowering in Arabidopsis
Source: BMC Plant Biol. 2014 Dec 30;14:381. doi: 10.1186/s12870-014-0381-5 (PMC4310184; doi:10.1186/s12870-014-0381-5)
Supplement: Additional file 6: Table S1. — Information on genes analyzed and primers used in this study. [file 12870_2014_381_MOESM6_ESM.pdf]

Additional File 6: Table S1. Information on genes tested and primers used in this study

Gene (locus code)	Locus code	Primers / product size (bp)
AP1	AT1G69120	5'-ATGCTGAAGTTGCTCTTGTTGT-3', 5'-AATCTTAGCCTTAAGCCTGTTA-3' / 188
EF1α	AT1G07920	5'-GCTGATTGTGCTGTCCTTAT-3', 5'-TAGTGGCATCCATCTTGTTAC-3' / 151
FD	AT4G35900	5'-TCCACCATTGTCACTGCTCT-3' 5'-TACGCTTATACCTTCTGTCTCC-3' / 220
METC 1	At3G03070	5'-AAAGCCCTAATCCGATCTCAGA-3' 5'-GGTGTCTCCTTCACAAGCAACA-3' / 210
METC 2	At5G67590	5'-ACAAGCGTGGTGAGATCGGTAA-3' 5'-AATCAAAAGCAAGAGCAGAGTC-3' / 228
METC 3	At2G02050	5'-GCACTTGGATCTAGAGATATGT-3' 5'-CGTTTCCTTGTAGTTTATTCTG-3' / 208
METC 4	At5G18800	5'-GATCCCTACTTCGGCGGTTTTA-3' 5'-CACCCAACATAATCATCCATCT-3' / 242
METC 5	At2G20800	5'-TTTTTCCTCTTCACTCCTCTCT-3' 5'-GTCAAACTCTGTTGTGCCCTTA-3' / 204
METC 6	At1G15120	5'-ATGGCAGATGATGAAGTTGTTG-3' 5'-TTTAGTTTCGCAAACAGCTTTG-3' / 206
METC 7	At3G10860	5'-AGCCGGTGAAATTGAAGGCGGT-3' 5'-GTGCTCAAGCTTCTCCTGTTCC-3' / 200
METC 8	At5G05370	5'-GGTGAAATTGAAGGCGGTGGTT-3' 5'-ACCTGTGCTCCAGCTTCTCTTG-3' / 200
METC 9	AT1G53030	5'-CACAAGACAGTGCGTGTTCTTT-3' 5'-CAAACTTTGAAACCCTCTGATC-3' / 202
METC 10	At5G04750	5'- TCGGCTCGTTCTGCGATCACAA-3' 5'- CCAGTCTCTCCCTCTCCATT' / 194
METC 11	AT2G19680	5'-CATCAAAATTGGTTCAACTTCA-3' 5'-CTTTCCTAAATGTTTCGTAGCG-3' / 210
METC 12	At4G29480	5'-TGCCACTGTTGAGAAGTGCCAA-3' 5'-TGTGAAGGTGAATCCTCTGCCG-3' / 232
METC 13	At1G51650	5'-TCGAATGCGGCGGTTCCGTTCT-3' 5'-TCAGGTGTGTCTGACCGCAAAA-3' / 200
RfBP	LOC102455463	5'- CGACTCCTCCTGCCTCTGCTT-3' 5'- ACCTCCACCCCTTCCTCTTCC-3'/ 171
